# Comparison Study of Oral Iron Preparations Using a Human Intestinal Model

**DOI:** 10.3797/scipharm.1304-03

**Published:** 2013-06-21

**Authors:** Mohammed Gulrez Zariwala, Satyanarayana Somavarapu, Sebastien Farnaud, Derek Renshaw

**Affiliations:** 1 Department of Human & Health Sciences, School of Life Sciences, University of Westminster, 115 New Cavendish Street, London, W1W 6UW, UK.; 2 Department of Pharmaceutics, UCL School of Pharmacy, 29–39 Brunswick Square, London, WC1N 1AX, UK.; 3 Department of Life Sciences, University of Bedfordshire, Park Square, Luton, LU1 3JU, UK.

**Keywords:** Anaemia, Dissolution, Caco-2, Ferritin, Iron Supplements

## Abstract

Iron deficiency and related iron deficiency anaemia (IDA) are the most prevalent nutritional disorders worldwide. The standard treatment involves supplementation with solid or liquid iron supplement preparations, usually based on a ferrous salt such as ferrous sulphate, ferrous fumarate, or ferrous gluconate. In the present study, we compared iron uptake and absorption from various solid and liquid iron supplement preparations currently available in the United Kingdom using the well-characterised human epithelial adenocarcinoma cell line Caco-2. Intracellular ferritin protein formation by the Caco-2 cell was considered an indicator of cellular iron uptake and absorption. We investigated the effects of formulation ingredients at a defined pH on iron uptake and absorption, and designed a novel two-stage dissolution-absorption protocol that mimicked physiological conditions. Our experiments revealed wide variations in the rate of dissolution between the various solid iron preparations. Conventional-release ferrous iron tablets dissolved rapidly (48 ± 4 mins to 64 ± 4 mins), whereas modified-released tablets and capsules took significantly longer to undergo complete dissolution (274 ± 8 to 256 ± 8 mins). Among the solid iron preparations, ferrous sulphate conventional-release tablets demonstrated the highest iron absorption, whereas modified-release ferrous preparations demonstrated uniformly low iron absorption, as compared to the control (P < 0.05). Taken together, our results demonstrate that there are wide-ranging variations in dissolution times and iron uptake from oral iron preparations, with the physical characteristics of the preparation as well as the form of iron playing a key role.

## Introduction

Iron is an essential mineral nutrient that has a key physiological role and is required for numerous functions such as oxygen transport, ATP production, and DNA replication. In normal healthy humans, the main source of iron loss is through shedding of skin and sloughed gastrointestinal mucosal cells [1]. In males this accounts for approximately 1 mg/day, while it is higher in women due to menstruation-associated blood loss. Iron cannot be synthesised in the human body and is therefore acquired primarily from dietary sources. Assuming a normal human diet, 12–18 mg of iron is ingested daily, mostly in the ferric form (Fe^3+^) [2]. Due to physiological as well as dietary factors, only 1–2 mg of this amount undergoes absorption through the gut enterocyte to become available to the systemic circulation [2].

During the digestion of food in the stomach, bound iron is liberated from its matrix via a combination of factors that include gastric acidity, enzymatic action, and the churning action of the stomach muscles established by the specialised oblique muscle layer, which is unique to the stomach [3]. The released iron is then available for absorption, which occurs predominantly in the duodenal segment of the small intestine [4].

Most dietary non-haem iron is in the insoluble ferric form which is first reduced to ferrous iron by the ferric reductase DcytB (duodenal cytochrome b) located on the brush border surface of duodenal enterocytes [5]. Divalent proton-coupled metal iron transporter (DMT1) then transports ferrous iron across the apical membrane into the enterocyte, where it is either complexed within the storage protein ferritin, or transported into circulation across the basolateral membrane via the transmembrane transporter ferroportin (Ireg1) [6]. Haem iron uptake occurs via an as yet unclarified mechanism which may involve the folate channel HCP1 [7]. The absorption process is sensitive to various factors, dietary and otherwise, that may impede this process leading to insufficient absorption and consequent iron deficiency [8].

According to the WHO, iron deficiency is the most common nutritional disorder affecting as much as 20% of the global population [9]. It arises when the body’s iron requirements are not met by dietary iron. Reduced iron delivery to target sites such as the liver parenchyma, bone marrow, and muscle myoglobin results in an impairment of iron-dependent functions such as erythropoiesis [1]. A decrease in the number of red blood cells may also be characterised by a smaller mean cell size (microcytic anaemia) [10]. The net outcome is decreased oxygen carrying capacity and consequent tissue hypoxia. Iron depletion and deficiency in its mildest form is not particularly detrimental, however, progression to iron deficiency anaemia (IDA) or sideropenic anaemia can have severe physiological consequences [11]. IDA during pregnancy has particularly severe consequences and has been associated with preterm delivery, perinatal mortality, maternal postpartum depression, and impaired mental development and cognitive ability of the offspring [12].

Treatment for iron deficiency and IDA is generally by means of oral iron supplements. Supplementation therapy can also be prescribed prophylactically in certain cases, e.g. for pregnant women, haemodialysis patients, and low birth weight infants. Intravenous iron administration is carried out in extreme cases such as significant blood loss or advanced malnutrition [13]. Oral iron supplementation preparations are available either as licensed preparations available under prescription by a medical practitioner as well as non-prescription over the counter (OTC) preparations, or as dietary or food supplements. Ferrous sulphate (FeSO_4_), along with the ferrous salts, ferrous fumarate and ferrous gluconate, are the most common form of iron supplements currently in use [14]. In the United Kingdom (UK), ferrous sulphate tablets (200 mg), usually containing 65 mg of elemental iron, are most often the first choice for treatment of iron deficiency anaemia [14]. Iron supplements are presented either as solid or liquid preparations with several possible variations.

In this study, we have used a modified dissolution absorption *in vitro* model developed in our laboratory which utilises the human intestinal Caco-2 cell to compare iron uptake absorption from various solid and liquid iron supplementation preparations that are currently available. To our knowledge, such a study has not been conducted previously.

## Materials and Methods

### Materials

All chemicals were of the highest available purity grade or cell culture grade, and purchased from Sigma-Aldrich (Dorset, UK) unless otherwise stated. Caco-2 cells were purchased from European Collection of Cell Cultures (Catalogue no. 09042001, ECACC, Salisbury, UK). Ferritin ELISA kits (Product code S-22) were from Ramco (ATI Atlas, Chichester, UK) and the BCA protein assay kit (Product no. 23225) was from Pierce (Thermo Fisher Scientific, Northumberland, UK). Protease inhibitor cocktail (PIC, catalogue no. P8340) was from Sigma-Aldrich (Dorset, UK). Cell culture media, foetal calf serum (FCS), and reagents were from Invitrogen (Loughborough, UK) and Lonza (Slough, UK). Cell culture plates (6-well and 96-well) and flasks were from Nunc (Roskilde, Denmark). All other cell culture plasticwares were supplied by Corning (Amsterdam, The Netherlands). Solid and liquid iron preparations from the UK were purchased from Boots retail pharmacy (London, UK). A summary of all samples tested is provided in Table 1. All reagents used were prepared using ultrapure water (resistivity of 18.2 MΩ cm). Prior to use, all glassware and utensils was soaked in 10% hydrochloric acid (HCl) and rinsed with ultrapure water to remove any potential traces of residual minerals.

### Methods

#### Cell Culture

Caco-2 cells were obtained at passage 20 and used experimentally between passages 30 to 35. Stock cultures were maintained in 75 cm^2^ tissue culture flasks in complete medium (Dulbecco’s modified Eagle’s media (DMEM) - Glutamax®, pH 7.4 supplemented with 10% FCS, 1% antibiotic/antimycotic solution and 25 mM HEPES). The cells were cultured in an incubator at 37°C in an atmosphere of 95% air and 5% CO_2_ at constant humidity, replacing the medium every two days. Cells were seeded onto 6-well plates at an initial seeding density of 1 × 10^4^ cells/cm^2^ for the dissolution absorption experiment. Parallel 6-well plates were also seeded similarly for the assessment of cell viability prior to the commencement of the uptake experiment and following completion. Caco-2 cells were differentiated to a fully matured gastrointestinal (GI) tract phenotype at day 14–15 post-seeding, at which time iron uptake experiments were commenced.

#### In vitro Dissolution

Dissolution conditions were simulated *in vitro* to replicate physiological conditions as closely as possible. Iron preparations were either solid (tablets or capsules) or liquid (syrup-based solutions) dosage forms. Tablets and capsules were further classified as conventional-release or modified-release as per formulation characteristics. HCl 0.1 M (pH 1.2) pre-warmed to 37°C was used to simulate gastric conditions, and a phosphate buffer adjusted to pH 5.8 with 2-(*N*-Morpholino)ethanesulfonic acid (10 mM) pre-warmed to 37°C was used to simulate intestinal conditions. On the day of the experiment, the solid dosage forms were placed into glass beakers containing 50 mls of simulated gastric or intestinal fluid, and stirred gently at 25 revolutions per minute (rpm) to simulate the peristaltic movements in the GI tract. Temperature was kept constant at 37°C. Liquid dosage forms were also added to the dissolution media solution and subjected to similar conditions. Aliquots from the dissolution media were withdrawn following complete dissolution (determined by visual monitoring) and used for the iron uptake experiment.

#### In vitro Iron Release

Iron release from the dosage forms was determined by carrying out dissolution experiments under the conditions specified in Ph.Eur.4/USP 26. Dissolution media (37°C, pH 1.2) was prepared as described in the previous section. Iron release was evaluated by withdrawing 1 ml aliquots at predetermined sampling intervals (15, 30, 60, 90, 120 mins) and replacing with the equivalent volume of fresh dissolution media. The sample aliquots were centrifuged at 13,000 rpm for 10 minutes at 4 °C and the supernatants were analysed for iron content.

#### Iron Quantification

Iron release was quantified using a modified ferrozine assay (15) developed in our laboratory. Briefly, sample aliquots (10 μl) were added to microcentrifuge tubes containing 300 μl HCL (0.5 M). Iron standards were prepared using analytical grade FeSO_4_. Detection reagent (500 μl containing ferrozine 2 mM, neocuproine 2 mM) was added to all tubes. The tubes were covered with aluminium foil and incubated in the dark for 60 mins after which time equal volumes (280 μl) of the test and standard samples were pipetted into a 96-well microplate in triplicate. Absorbance was read at 562 nm using a microplate reader (VersaMax, Molecular devices, USA) and the iron content in the test samples was calculated from the standard curve generated from the iron standards. Iron release (% iron content as per label claim) from the dosage forms was determined from these values.

#### Iron Uptake and Absorption by Caco-2 Cells

On day 13 post-seeding, growth media was aspirated, cells were washed three times with wash solution (140 mM NaCl, 5 mM KCl, 10 mM PIPES buffer, pH 6.7, 37°C), and were then incubated in serum-free MEM for 24 hours. On the day of the uptake experiments, test media were prepared by titrating MEM with 0.1 M HCl or 0.1 M NaOH to pH 5.8 (representative of the physiological pH in the duodenum). Test media was then sterile-filtered using a 0.2 μm filter unit and then buffered with 2-(*N*-Morpholino)ethanesulfonic acid (MES, 10 mM). FeSO_4_ solution was used as a reference standard and was prepared by dissolving ultrapure FeSO_4_ powder in 0.1 M HCl. Media (14 ml) was aliquoted into individual falcon tubes and varying volumes of samples from the dissoluted test and reference preparations were added to the test media to achieve a final concentration of 20 μM elemental iron for each condition. The pH of the iron-enriched sample media was measured again and adjusted to 5.8, when required. The trypan blue exclusion assay was carried out in parallel 6-well plates prior to commencing the experiments to assess Caco-2 cell viability. Briefly, cells were stained with trypan blue dye which selectively stains dead cells; these were then counted under a microscope and the percentage viability was calculated. The Caco-2 cells in test plates were washed three times with wash solution and then incubated with iron-enriched test media (2 ml per well, three wells per condition) for 2 hours at 37°C in a plate incubator rocking gently at 25 rpm. The test media was then aspirated and the cells were washed twice with wash solution and finally with a removal solution (wash solution plus 5 μm Na hydrosulphite and 1 μm bathophenanthroline disulfonate) to remove any surface-bound iron [16]. Caco-2 cells were then incubated with fresh MEM for a further 24 hours in a cell culture incubator (37°C, 95% air and 5% CO_2_). Parallel 6-well plates were also incubated with test media (prepared as described above) and subjected to similar conditions (one well per condition). The Caco-2 cell viability in these plates was assessed at the completion of the experiment using trypan blue dye exclusion assay.

#### Caco-2 Cell Harvesting

The culture media was aspirated 24 hours after incubation and Caco-2 cells were washed twice with wash buffer. The cells were harvested by addition of 350 μl lysis buffer (50 mM NaOH supplemented with 1μg/ml protease inhibitor cocktail) per well for 40 minutes while rocking gently on a plate shaker (6 rpm). The temperature was standardised at 4 °C throughout the lysis protocol. Cells were then collected using a sterile cell scraper and the resultant lysate was pipetted into 0.5 ml microcentrifuge tubes. Each sample lysate was passed six times through a 1 ml syringe fitted with 25 gauge needles to reduce viscosity and ease sample handling. Samples were aliquoted into two equal batches and stored immediately at −20°C until further analysis.

#### Analytical Methods (Ferritin ELISA and BCA)

The total ferritin concentration of the cell lysates was determined using a spectro-photometric ELISA kit following the manufacturer’s protocol with minor modifications. Frozen cell lysates were centrifuged for 10 minutes (13226 *g*, 4°C) and the resultant supernatant was measured in the assay. A standard curve was generated using the standards provided (0, 6, 20, 60, 200 ng standard/ml). Samples and standards (30 μl each) were loaded in triplicate onto a 96-well plate and the incubation steps were carried out as described in the protocol. Absorbance was determined at 490 and 630 nm using a microplate reader (VersaMax, Molecular devices, USA). The protein content of the Caco-2 cells was determined using the Pierce BCA kit following the manufacturer’s protocol using the bovine serum albumin (BSA) stock (2 mg/ml) provided in the kit as the standard. All samples were assayed in duplicate. The ferritin concentration was standardised against the total protein concentration and ng ferritin/ mg protein was used as an indicator of iron uptake and absorption by the Caco-2 cells.

#### Statistical Analysis

Iron uptake experiments were carried out twice independently, with each treatment condition performed in triplicate. A mean of six replicates was calculated for each treatment. The data is presented as the mean ± SEM and the differences between samples were analysed via Tukey’s one-way ANOVA followed by Tukey’s *post hoc* test using Graphpad Prism software (Version 5.0). Results were considered significantly different if P < 0.05.

## Results

In this study, we compared the iron uptake from various oral iron preparations using an *in vitro* model that attempted to mimic physiological conditions. The most commonly prescribed classical iron preparations were evaluated.

We first carried out an experiment to compare dissolution rates of the various test preparations. Since we were investigating conventional- as well as modified-release preparations, the dissolution experiments were carried out at pH 1.2 to recreate the gastric environment, as well as at pH 5.8, to recreate the environment in the small intestine. At time zero, the tablets or capsules were dropped into the dissolution medium, and the dissolution runs for each preparation were carried out in triplicate. All preparations were taken from at least two different batches. The dissolution vessels were constantly monitored to observe the dissolution profiles of the test preparations. Complete dissolution of the tablet or capsule as observed visually was taken as the experimental endpoint. Results of the dissolution experiments are shown in Table 2.

**Fig. 1:**
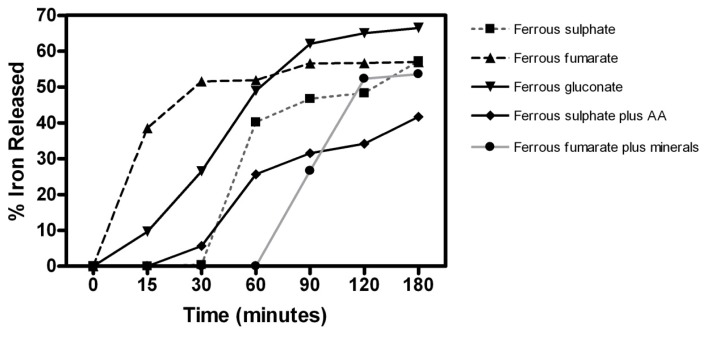
Iron release from oral iron preparations available in the United Kingdom. Dosage forms were subjected to *in vitro* dissolution (0.1M HCl pH 1.2, 37°C) and aliquots withdrawn at fixed intervals. Iron content in the sample aliquots was deteremined using a colorimetric iron quantification assay and used to calculate the % iron release. Values shown are mean of three readings for each test sample.

At pH 1.2, the conventional-release tablets exhibited a rapid rate of dissolution while the modified-release tablets and capsules dissolved gradually and variably. Ferrous sulphate tablets (unbranded/Almus) dissolved the fastest (48 ± 4 mins) followed by ferrous fumarate (Fersamal/Goldshield, 57 ± 6 mins) and ferrous gluconate tablets (unbranded/Zanza 64 ± 4 mins). The slowest rate of dissolution was demonstrated by ferrous fumarate sustained-release capsules (Feroglobin/Vitabiotics, 274 ± 8 mins) and ferrous sulphate controlled-release tablets (Ferrograd C/Teopharma, 256 ± 8 mins). Under simulated intestinal conditions (pH 5.8), the fastest dissolution time was observed for ferrous fumarate tablets (85 ± 9 mins), whereas ferrous fumarate controlled-release tablets (Ferrograd C/Teopharma) failed to dissolve completely even after 24 h. Ferrous fumarate sustained-release capsules (Ferroglobin B12/Vitabiotics) demonstrated a mean dissolution time of 388 ± 11 mins at pH 5.8. We also investigated time-dependent iron release characteristics from test preparations by carrying out a separate series of dissolution experiments under simulated gastric conditions. The iron release form test preparations at varying time points (15, 30, 60, 90, 180 mins) were quantified using a colorimetric assay. Results are shown below in Fig. 1. Initial iron release at the first time point (15 min) was highest from the ferrous fumarate tablets (38.5%) while iron release at the final time point (180 min) was highest from the ferrous gluconate tablets (66.46%; see Fig. 1).

Following the dissolution and release studies, we conducted iron uptake experiments. The trypan blue exclusion method was used to examine the Caco-2 cell viability prior to the commencement of the uptake experiments as well as following completion, to examine any detrimental effects of the formulation excipients on the Caco-2 cells. Viability above 90% was observed in all cases (Data not shown).

**Fig. 2:**
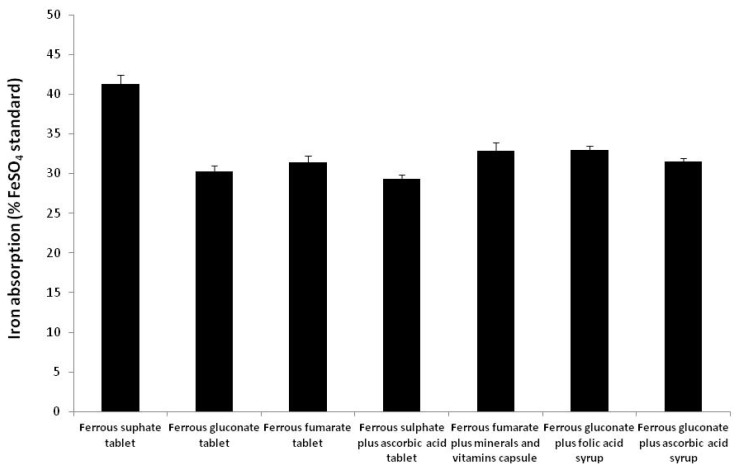
Iron absorption from oral iron preparations available in the United Kingdom. Caco-2 cells were incubated with 20 μM iron from each of the test preparations. Iron absorption in terms of ferritin (ng/mg total protein) was determined by ELISA. Results are expressed as a % of ferrous sulphate control which is considered as 100%. Results are means ± SEM of 6 samples for each condition (P<0.05). The values are representative of three independent experiments.

Our aim was to compare the diverse range of preparations currently available in the UK using a defined and uniform set of conditions. The three most commonly used ferrous salts; ferrous sulphate, ferrous gluconate, and ferrous fumarate were tested in the form of conventional-release tablets. Ferrous sulphate plus ascorbic acid (Ferrograd C) and ferrous gluconate plus minerals and vitamins (Feroglobin B12) were included as representative modified-release and multivitamin preparations. Ferrous gluconate plus folic acid (Feroglobin B12) and ferrous gluconate plus ascorbic acid (Floradix) were included as representative syrup-based liquid preparations. These samples include both prescription and OTC iron supplement preparations. Ultrapure FeSO_4_ powder was included as a reference standard and absorption results were standardised to those of FeSO_4_ (considered as 100%). Results are shown in Fig. 2.

Iron absorption from ferrous sulphate conventional-release tablets was significantly higher than any of the preparations evaluated (P<0.05), and equivalent to 41% of the FeSO_4_ standard. Both the modified-released formulations containing ferrous sulphate plus ascorbic acid (Ferrograd C) and ferrous gluconate plus minerals and vitamins (Feroglobin B12) demonstrated low iron absorption. Syrup preparations containing ferrous gluconate plus folic acid (Feroglobin B12) and ferrous gluconate plus ascorbic acid (Floradix) also demonstrated relatively low iron absorption. Conventional-release ferrous sulphate therefore demonstrated the overall highest rate of iron uptake, whereas the lowest level of iron uptake was from ferrous sulphate plus ascorbic acid (Ferrograd C) modified-release tablets.

## Discussion

Iron supplementation is considered an effective strategy to counter anaemia, and iron preparations are some of the most commonly prescribed drugs [17]. Ferrous salts, first used in the 19th century, have remained the mainstay of iron therapy [1, 18]. The main advantages are low unit cost, thereby rendering them widely accessible. However, ferrous salts have a high propensity to cause side effects which generally lead to low patient compliance. Diarrhoea and constipation are commonly encountered by approximately 6% of the patients, whereas 6–12% of the patients report nausea, vomiting, and gastric distress [19]. Most of these side effects are attributed to the fraction of iron that remains unabsorbed following ingestion and remains localised in the intestinal lumen. Some authors have attributed these side-effects to the irritation of the GI mucosa by the catalysis of free radicals from non-transferrin bound free iron [20].

In the present study, we compared iron absorption amongst several commercially available iron preparations. Representative products of the most commonly used iron preparations in the UK were evaluated in our study. We focused on these, as the preparations available in the UK undergo extensive regulatory procedures and also represent most iron preparations available globally. Very few studies *in vivo* or *in vitro,* however, have compared iron absorption from oral dosage forms and to our knowledge, no study has used a two-stage *in vitro* dissolution-absorption protocol.

Iron absorption takes place principally in the duodenum region of the small intestine, and the human intestinal cell line Caco-2 has been well-characterised as a model for studying iron absorption *in vitro*[21]. Caco-2 cells exhibit the morphological characteristics of mature enterocytes and express most of the receptors involved in iron absorption, including DMT-1, DcytB, and Ireg1 (ferroportin), IRE1, and IRE2. Several studies have previously compared iron uptake from iron salts, whole foods, fruit juices, and infant formulas using the Caco-2-ferritin quantification model [22–26]. Most oral iron supplement preparations are however administered as dosage forms (tablets, capsules, syrups, or solutions) and include a number of active and inactive ingredients (e.g. binders, diluents, disintegrants) necessary for the formulation that may potentially have an influence on iron uptake and absorption. Active ingredients may include other minerals and vitamins such as ascorbic acid (vitamin C), which is often included in combination with iron as it is generally regarded as an enhancer of iron absorption [27]. In conditions that reflect a need for replacement therapy, such as during pregnancy and malnutrition, experts also recommend iron in combinations with other micronutrients such as folic acid and vitamin B12 [28]. In the case of liquid preparations such as syrups, additives such as flavourants and colourants are also added to increase presentation and palatability. Several iron syrup, solution, and elixir preparations contain fruit and plant juices or extracts. Fruit juices and vegetable extracts are rich in polyphenols; known inhibitors of iron absorption [29–31]. We therefore wanted to investigate the combined effect of the physical form of the preparation in combination with the iron source.

Following ingestion, solid oral formulations undergo dissolution under the influence of the acidic pH of the stomach assisted by the peristaltic motion and segmentation of GI muscles. Dissolution is the rate-determining step for the absorption of various oral drugs, and dissolution rates vary widely, depending upon the nature of the formulation [32]. Fast-dissolving tablets release iron quickly; however, this might lead to side effects due to excess free iron localisation/accumulation and consequent chemical irritation of the GI mucosa [33]. To counter this, slow- or modified-release tablets have been formulated. Modified-release tablets or capsules are pharmaceutical strategies to maintain a slow and gradual release of the active ingredient following ingestion, based on the rationale that iron released in such a manner is better tolerated as it would avoid overaccumulation and its consequent adverse effects. This is usually achieved by coating the tablet with a film, or by entrapping the active ingredient in a polymeric matrix, thereby causing it to release at a slow and gradual rate. Such iron preparations are relatively more expensive than conventional tablets [33]. The physiological process of gastric emptying limits the relative amount of time that tablets and other solid dosage forms have available to them to undergo complete dissolution. Gastric emptying time for adult humans is between 3–6 h [34], and therefore if the tablet does not undergo complete dissolution within this period the residual undissolved fraction will be transported into the large intestine to be subsequently excreted. This would imply loss of a major proportion of the dosage form before the active ingredient can be fully released and available for absorption [35]. Regulatory bodies such as the UK National Health Service (NHS) have therefore recommended against the use of modified-release iron formulations [33].

At present, there is no dissolution test indicated for iron preparations as per the British Pharmacopoeia (BP), while the United States Pharmacopoeia (USP) specifies a dissolution test only for ferrous gluconate tablets and capsules [36]. We developed a two-step dissolution-absorption protocol that aims to replicate the temperature, pH, acidity, and mechanical conditions that the dosage forms encounter within the human stomach. Our protocol takes into consideration the effect of dissolution time, excipients, and variations in pH on iron absorption from oral preparations.

Prior to our experiments, we carried out optimisation studies to evaluate dissolution time for all solid preparations being tested. HCL (0.1 M, pH 1.2) was used to simulate gastric fluid to mimic the pH environment of the stomach, and a phosphate buffer (pH 5.8) was used to simulate intestinal fluid to take into account the dissolution pattern of modified-released preparations. Conventional fast-release tablets were found to dissolute completely in ~2 h (under gastric pH conditions), whereas modified-release tablets took significantly longer (~4 h). At intestinal pH conditions, the modified-release dosage forms demonstrated variable dissolution rates, with the controlled-release tablet Ferrograd C failing to undergo complete dissolution even after 24 hours. Our data is in agreement with a study by Bannerman and colleagues where dissolution profiles of various solid oral iron preparations were compared [36].

We also examined iron release from the dosage forms during the dissolution process, and the results corresponded to our dissolution data. Our results indicated that the iron preparations appear to follow the predicted pattern of drug release as per their dosage form design i.e. the conventional-release tablet demonstrated rapid iron release while the controlled- and sustained-release formulations demonstrated a gradual release pattern over a longer period.

After the iron preparations were subjected to dissolution, aliquots were withdrawn, the equivalent of 20 μM Fe final concentration, and were used for the iron uptake experiments. This concentration was selected based on optimisation experiments carried out in our laboratory, and is in agreement with several previous studies that have found this concentration to be optimum for iron uptake studies [25, 37].

Excipients are known to play either a facilitatory or inhibitory a role on drug absorption [38]; we therefore did not filter the samples, thus allowing us to study the effects of excipients on iron uptake in the Caco-2 cells. The pH of the treatment media was adjusted to pH 5.8, which is a representative pH in the distal duodenum and has been optimised by others [39]. Uptake experiments were standardised for 2 hours, which represents the physiological transit time through the small intestine [40].

We measured intracellular ferritin concentrations as a measure of iron absorption in Caco-2 cells. This method is very sensitive, has been well-characterised, and shows a good correlation with human absorption. Caco-2 cells synthesise ferritin in response to their iron status, as well as iron levels in their surrounding environment [37]. We therefore cultured the cells in media containing minimal amounts of iron 24 hours prior to the experiment to ensure minimal cellular ferritin formation prior to the uptake experiments. Under these conditions, Caco-2 cells demonstrate maximal iron uptake and therefore minute variations in iron uptake between the various samples can be measured accurately in terms of the ferritin formation [21]. Iron uptake studies can also be carried out by measuring intracellular total iron uptake using either atomic absorption spectroscopy or radio-labelled iron. These methods necessitate the requirement for complex instrumentation and specialised facilities. Some iron compounds and complexes cannot be labelled either intrinsically or extrinsically with iron radioisotopes. Ferritin measurement avoids these issues and also circumvents the problem of non-specific binding of free iron to the cell surface that might lead to inaccurate total cellular iron quantification.

Our study has demonstrated that amongst the various iron preparations tested, conventional fast-release ferrous sulphate tablets demonstrate the highest iron absorption, whereas modified-release iron tablets uniformly had the lowest iron absorption. This is in agreement with other studies that have also shown modified-release or iron formulations to have poor bioavailability [36, 40]. Equal doses of elemental iron (20 μm) from each preparation were used in our uptake experiments, thus the variations in iron absorption between the various preparations cannot be attributed to the differences in iron content of each formulation. A possible explanation for the variability in iron absorption between the samples may be due to the formulation characteristics of the iron preparation form. Conventional-release iron tablets are formulated to release iron rapidly and contain fewer excipients. Modified-release preparations are relatively more complex preparations and include several excipients and additives. Certain excipients are included to enhance absorption of the active ingredient [41]. However, excipients can also have a detrimental effect on uptake and absorption, by entrapping the active ingredient in a matrix, thereby making it unavailable for cellular uptake [42, 43]. The liquid iron preparations tested are also formulated with numerous excipients, mainly to improve palatability and appearance. Certain additives present in these preparations are known to inhibit iron absorption. For example, the liquid preparation Floradix is formulated in a syrup base that includes 29% fruit juice including grape and cherry juice, which may inhibit iron absorption due to their high polyphenol content [31]. Free FeSO_4_ solution, included as a reference standard in our experiments, demonstrated the highest iron uptake. Our results therefore indicate that the physical forms of the preparation, as well as the iron form, have an influence on iron uptake and absorption from oral dosage forms.

Comparison of iron absorption from oral dosage forms can be a valuable tool in assessing the efficacy of oral iron preparations. Iron supplementation is considered a convenient and economical measure to counter iron deficiency and its consequences, and a wide variety of solid and liquid iron preparations are available either as prescription or OTC supplement products. This study did not seek to challenge the fundamental strategy, but rather to explore any variations in iron release and uptake that might exist between the various preparations that could lead to less favourable outcomes of iron supplementation therapy. We believe that this study provides important insights into the physiological process of iron absorption from oral iron preparations and may therefore assist in the development of novel preparations that deliver requisite amounts of iron, while limiting adverse effects. Our future studies will determine iron absorption from oral preparations in the presence of food substances and will evaluate correlations with clinical data.

## Figures and Tables

**Tab. 1:** List of oral iron supplement preparations compared for dissolution testing and iron absorption. Test samples include solid and liquid preparations currently available in the United Kingdom.

No	Preparation	Dosage from	Trade name / Manufacturer	Elemental Fe/dos (mg)	Category
1	Ferrous sulphate	Tablet	NA Almus	65	Licenced/ non proprietary
2	Ferrous fumarate	Tablet	Fersamal Goldshield	68	Licenced/ non proprietary
3	Ferrous gluconate	Tablet	NA Zanza	35	Licenced/ proprietary
4	Ferrous sulphate plus ascorbic acid	Controlled release tablet	Ferrograd C Teopharma	105	Licenced/ non proprietary
5	Ferrous fumarate plus minerals and vitamins	Sustained release capsule	Ferroglobin B12 Vitabiotics	24	Over the counter
6	Ferrous gluconate plus folic acid	Syrup	Ferroglobin Vitabiotics	7.5	Over the counter
7	Ferrous gluconate plus ascorbic acid	Syrup	Floradix Salus	10	Over the counter

**Tab. 2:** Mean dissolution time for oral iron preparations. Dissolution time (mins) for solid oral iron preparations was tested in simulated stomach (0.1 M HCL pH 1.2, 37°C) and simulated intestinal (Phosphate buffer, pH 5.8, 37°C) conditions. Values are mean ± SEM (n = 3).

No	Preparation	Dosage from	Trade name / Manufacturer	Dissolution time (mins, n=3)	

				pH 1.2	pH 5.8
1	Ferrous sulphate	Tablet	NA Almus	48 ± 4	362 ± 16
2	Ferrous fumarate	Tablet	Fersamal Goldshield	57 ± 6	85 ± 9
3	Ferrous gluconate	Tablet	NA Zanza	64 ± 4	208 ± 12
4	Ferrous sulphate plus ascorbic acid	Controlled release tablet	Ferrograd C Teopharma	256 ± 8	–
5	Ferrous fumarate plus minerals and vitamins	Sustained release capsule	Ferroglobin B12 Vitabiotics	274 ± 8	388 ± 11

## Abbreviations

DMT1: Divalent proton-coupled metal iron transporter
Dcytb: Duodenal cytochrome b
IDA: iron deficiency anaemia
NHS: National Health Service
OTC: over the counter
